# Lipoxin A_4_ Attenuates the Inflammatory Response in Stem Cells of the Apical Papilla via ALX/FPR2

**DOI:** 10.1038/s41598-018-27194-7

**Published:** 2018-06-11

**Authors:** A. Gaudin, M. Tolar, O. A. Peters

**Affiliations:** 1 Department of Endodontics, University of Nantes, Nantes, France; 2 Centre de Recherche en Transplantation et Immunologie UMR1064, INSERM, Université de Nantes, Nantes, France; 3 Department of Orthodontics, University of the Pacific School of Dentistry, CA San Francisco, USA; 4 Department of Endodontics, University of the Pacific School of Dentistry, CA San Francisco, USA; 5 University of Queensland, School of Dentistry, Oral Health Centre, Herston, QLD Australia

## Abstract

Similar to the onset phase of inflammation, its resolution is a process that unfolds in a manner that is coordinated and regulated by a panel of mediators. Lipoxin A4 (LXA_4_) has been implicated as an anti-inflammatory, pro-resolving mediator. We hypothesized that LXA_4_ attenuates or prevents an inflammatory response via the immunosuppressive activity of Stem Cells of the Apical Papilla (SCAP). Here, we report for the first time *in vitro* that in a SCAP population, lipoxin receptor ALX/FPR2 was constitutively expressed and upregulated after stimulation with lipopolysaccharide and/or TNF-α. Moreover, LXA_4_ significantly enhanced proliferation, migration, and wound healing capacity of SCAP through the activation of its receptor, ALX/FPR2. Cytokine, chemokine and growth factor secretion by SCAP was inhibited in a dose dependent manner by LXA_4_. Finally, LXA_4_ enhanced immunomodulatory properties of SCAP towards Peripheral Blood Mononuclear Cells. These findings provide the first evidence that the LXA_4_-ALX/FPR2 axis in SCAP regulates inflammatory mediators and enhances immunomodulatory properties. Such features of SCAP may also support the role of these cells in the resolution phase of inflammation and suggest a novel molecular target for ALX/FPR2 receptor to enhance a stem cell-mediated pro-resolving pathway.

## Introduction

The role of inflammation in tissue regeneration is multi-faceted. According to current thinking, early pro-inflammatory signaling is detrimental while anti-inflammatory signaling may be beneficial for stem cell activity^1^. In the presence of an inflammatory environment (*e*.*g*., high levels of tumor necrosis factor α (TNF-α) and interferon-γ (IFN-γ)), mesenchymal stem cells (MSC) may be activated and assume immunosuppressive functions by secreting high levels of anti-inflammatory soluble factors (*e*.*g*., indoleamine-pyrrole 2,3-dioxygenase, prostaglandin E2, nitric oxide, transforming growth factor β, heme-oxygenase^2^). In the absence of an inflammatory environment (*i*.*e*., low levels of TNF-α and IFN-γ), MSC adopt a pro-inflammatory phenotype, thus enhancing the immune response by secreting chemokines that recruit leukocytes to a site of tissue injury (*e*.*g*., chemokine ligands such as CCL3, 4, 5, CXCL9, and CXCL10)^3,4^.

Similar to the onset phase of inflammation, its resolution is coordinated and regulated by a panel of mediators including specialized pro-resolving lipid mediators. These mediators are derived from polyunsaturated fatty acids and include lipoxins, maresins, resolvins and protectins^5^.

Lipoxin A_4_ (LXA_4_), for example, is a pro-resolving mediator secreted by immune cells such as neutrophils and macrophages. Lipoxins regulate functions of the innate immune system and also modulate the adaptive immune system by decreasing memory B-cell responses^6^. These actions are mediated by the activation of ALX/FPR2 receptor, a specific G-protein-coupled receptor that binds LXA_4_ with high affinity^7^.

Stem Cells from the Apical Papilla (SCAP) are dental mesenchymal stem cells characterized by their pluripotency and ability to differentiate into several cell-restricted lineages^8^. Under specific conditions *in vitro*, SCAP are reported to differentiate in functional dentinogenic cells, which are capable of producing typical dentin-like structures^9,10^; these findings suggest that SCAP could be a potential source of odontoblast progenitors. Similar to bone marrow mesenchymal stem cells, SCAP appear to possess immunomodulatory properties. For example, SCAP are able to inhibit T-cell proliferation in a mixed lymphocyte reaction^11^. Cell-cell contact and/or paracrine mechanisms are putatively involved, however, the exact mechanism of this interaction is currently unknown. Under clinical conditions, reparative efforts of odontoblast-like cells occur in inflammatory conditions. Therefore, we hypothesized that LXA_4_ may attenuate or prevent the inflammatory response via the immunosuppressive activity of SCAP. The aim of our study, therefore, was to investigate interactions of LXA_4_ with SCAP, while focusing on the role of LXA_4_ binding to ALX/FPR2 receptor.

## Results

### SCAP maintain stem cell properties and share similar surface markers with periodontal ligament cells (PDLC)

**Figure 1:**
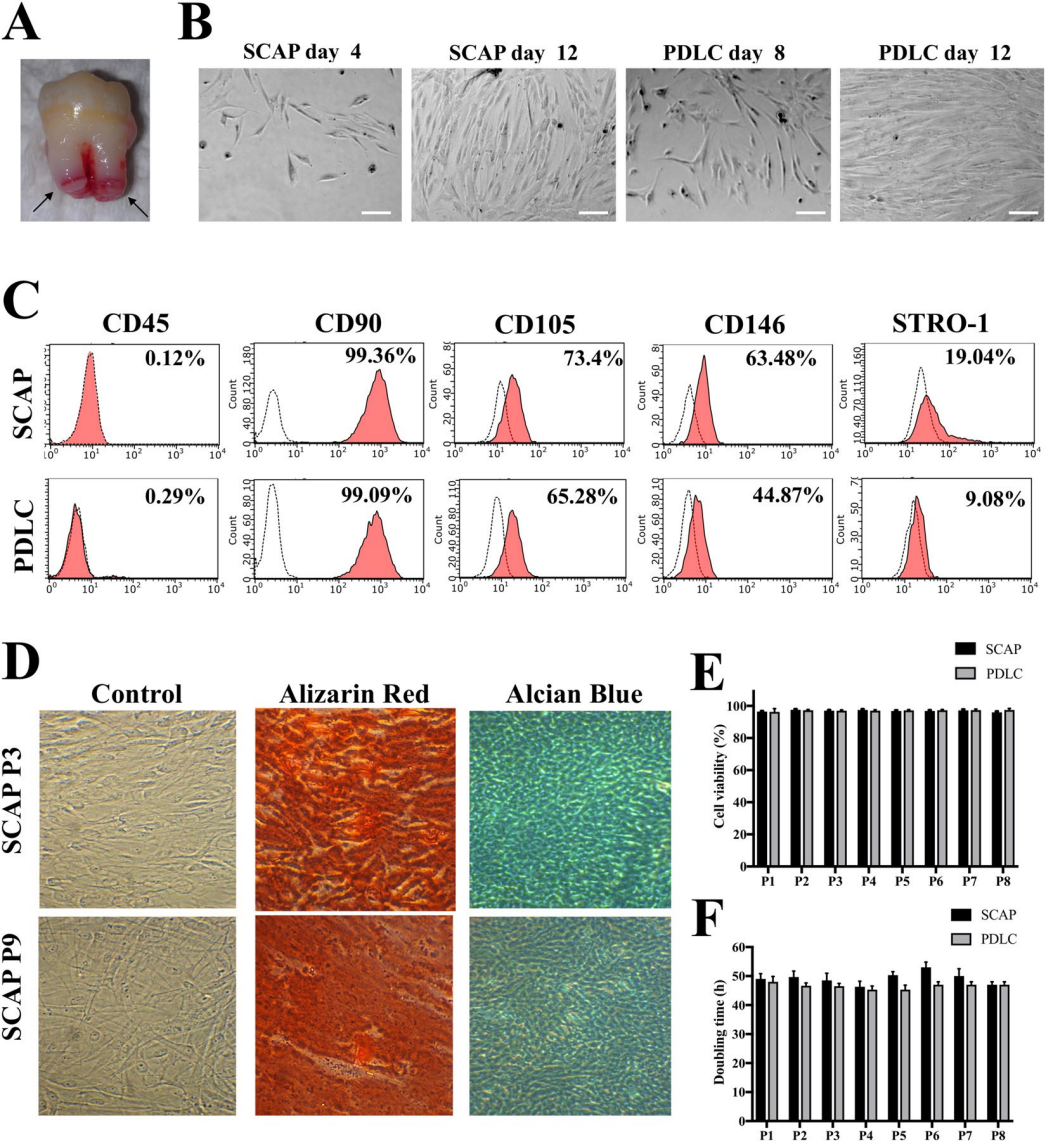
Characterization of stem cells of the apical papilla (SCAP) in comparison with periodontal ligament fibroblast (PDLC). (**A**) Freshly extracted human third molar. The arrows indicate apical papilla tissue of immature tooth. (**B**) Generation of fibroblast colonies from single cells after 8 to 12 days of culture. Representative phase contrast microscopic photographs of generation and expansion of SCAP and PDLC. Cells have elongated shapes and grow attached to substrata. Scale bar, 25 μm. (**C**) Flow cytometry analysis of representative histograms at passage 3 (P3) showed that SCAP and PDLC expressed cell surface human mesenchymal stem markers (CD90, CD105, CD146 and STRO-1) and lacked the expression for leukocyte common antigen (CD45) (in red) compared with their appropriate isotype controls (dash line). (**D**) Differentiation of SCAP to odontoblast-like and chondrocyte-like phenotype. Unsorted SCAP at passage 3 and 9 were subjected to differentiation media for 2 weeks, which resulted in deposits positive for alizarin red and alcian blue stain, respectively. (**E**) The cell viability (trypan blue exclusion assay) of SCAP and PDLC was stable and similar from P1 to P8 for both populations. (**F**) Cell doubling times were stable and similar from P1 to P8 for both populations.

SCAP and PDLC were isolated from different donors (Fig. 1A). Both cell populations presented typical homogeneous fibroblast-like morphology and were able to generate fibroblast-like colonies from single cells after 8 to 12 days of culture (Fig. 1B). Morphological features of fresh and post-thaw cells were similar. To characterize the SCAP population *in vitro*, we performed flow-cytometric analysis of mesenchymal stem cell surface markers. Both SCAP and PDLC expressed specific MSC antigens (CD90, CD105, CD146 and STRO-1) and were negative for a hematopoietic marker, CD45 (Fig. 1C). Next, we sought to determine whether SCAP would undergo osteogenic and chondrogenic differentiation. Under osteo-inductive conditions for 3 weeks, SCAP produced mineralized extracellular matrix that was stained with alizarin red. After 21 days in chondrogenic conditions, deposition of chondrogenic-like matrix was revealed by alcian blue staining. Results were essentially the same at different passages (3 and 9) (see Fig. 1D). To demonstrate any impact of passaging on SCAP and PDLC viability and on expansion capacity, a trypan blue exclusion test was done and population doubling times were calculated. Cell viability in SCAP and PDLC was comparable and high from passages 1 to 8 (Fig. 1E). Cell doubling time was stable (around 48 h) and similar between SCAP and PDLC from passage 1 to 8 (Fig. 1F). In order to obtain more purified populations, SCAP sorted with immunomagnetic separation using STRO-1, CD73, CD90 and CD105 were characterized and compared with non-purified (mixed) SCAP and human gingival fibroblasts (Supplementary File S1). There was no effect of SCAP passages on *in vitro* differentiation and cell surface markers (Supplementary File S1A,C. ALX/FPR2 has been recently identified in PDLC^12^. Thus, in our study, we used PDLC as a positive control in the experiments.

### SCAP normally express ALX/FPR2 and this receptor is overexpressed when inflammatory stimuli are applied

**Figure 2:**
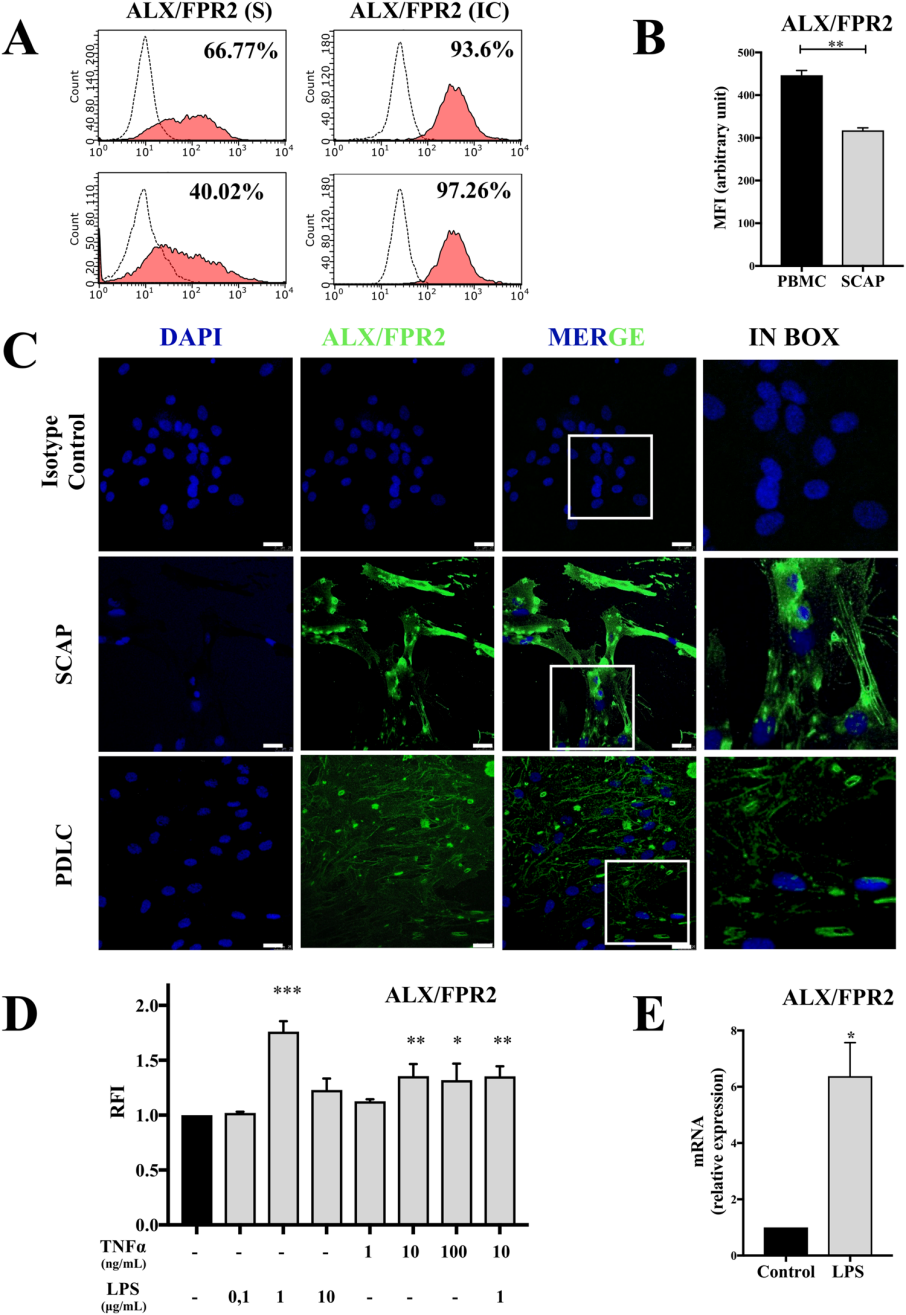
Expression of formyl peptide receptor 2 (ALX/FPR2) in SCAP is upregulated under inflammatory condition. (**A**) Flow cytometry analysis of representative histograms at passage 3 (P3) showed that SCAP and PDLC expressed surface (S) and intracellular (IC) ALX/FPR2. ALX/FPR2 antibody (red) and secondary antibody staining with appropriate isotype controls (dash line) (n=6). (**B**) Quantification of ALX/FPR2 expression by flow cytometry analysis shown as MFI (Mean Fluorescence Intensity) in SCAP and Peripheral Blood Mononuclear Cells (PBMC). **p < 0.01. (**C**) Representative confocal images of ALX/FPR2 distribution in permeabilized SCAP and PDLC. No immunostaining was observed in control conditions with an isotype control. Anti-ALX/FPR2 (green), nuclei (blue). Original magnification 43x (n=4). (**D**) 1 μg/mLof LPS and highest dose of TNF-α upregulated expression of ALX/FPR2 in SCAP at 24h. The expression of ALX/FPR2 was analyzed by flow cytometry using RFI (Relative Fluorescence Intensity). RFI presented here as a ratio between median fluorescence intensity (MFI) from experimental groups (grey histograms) and MFI from control group (black histogram). ***p < 0.001 versus control. (**E**) Lipopolysaccharide (LPS) stimulation of SCAP led to a significant increase of ALX/FPR2 mRNA expression compared to control after 24h. ALX/FPR2 mRNA expression was quantified by RT-PCR. *p < 0.05 versus control.

In order to explore the roles of the LXA_4_-ALX/FPR2 axis in SCAP, we investigated the expression of ALX/FPR2 under resting and simulated inflammatory conditions. To demonstrate the surface and intracellular expression of ALX/FPR2, we used flow cytometry of intact and permeabilized cells. Intracellular expression of ALX/FPR2 was higher than surface expression. PDLC and Human peripheral blood mononuclear cells (PBMC) were used as positive controls (Fig. 2A,B). Confocal microscopy confirmed expression at the protein level of ALX/FPR2 in SCAP (Fig. 2C). Then, we investigated the effect of various doses of two inflammatory factors (TNF-α and lipopolysaccharide (LPS)) on ALX/FPR2 expression using flow cytometry. We found that 1 μg/mL of LPS had a maximal inductive effect in SCAP at 24 hours, as shown by flow cytometric analysis. Only the highest dose (10 and 100 ng/mL) of TNF-α upregulated the expression of ALX/FPR2 in SCAP at 24 hours. We showed that TNF-α (10 ng/mL) in combination with LPS (1 μg/mL) also upregulated expression of ALX/FPR2 in SCAP at 24 h, but to a lesser degree than LPS alone (1 μg/mL) (Fig. 2D).

Finally, we confirmed by RT-PCR that SCAP express ALX/FPR2. Moreover, SCAPs significantly boosted ALX/FPR2 mRNA expression after they were incubated in LPS (1 μg/mL) for 24 h (Fig. 2E).

### LXA_4_ enhances proliferation and migration of SCAP via ALX/FPR2 receptor

**Figure 3:**
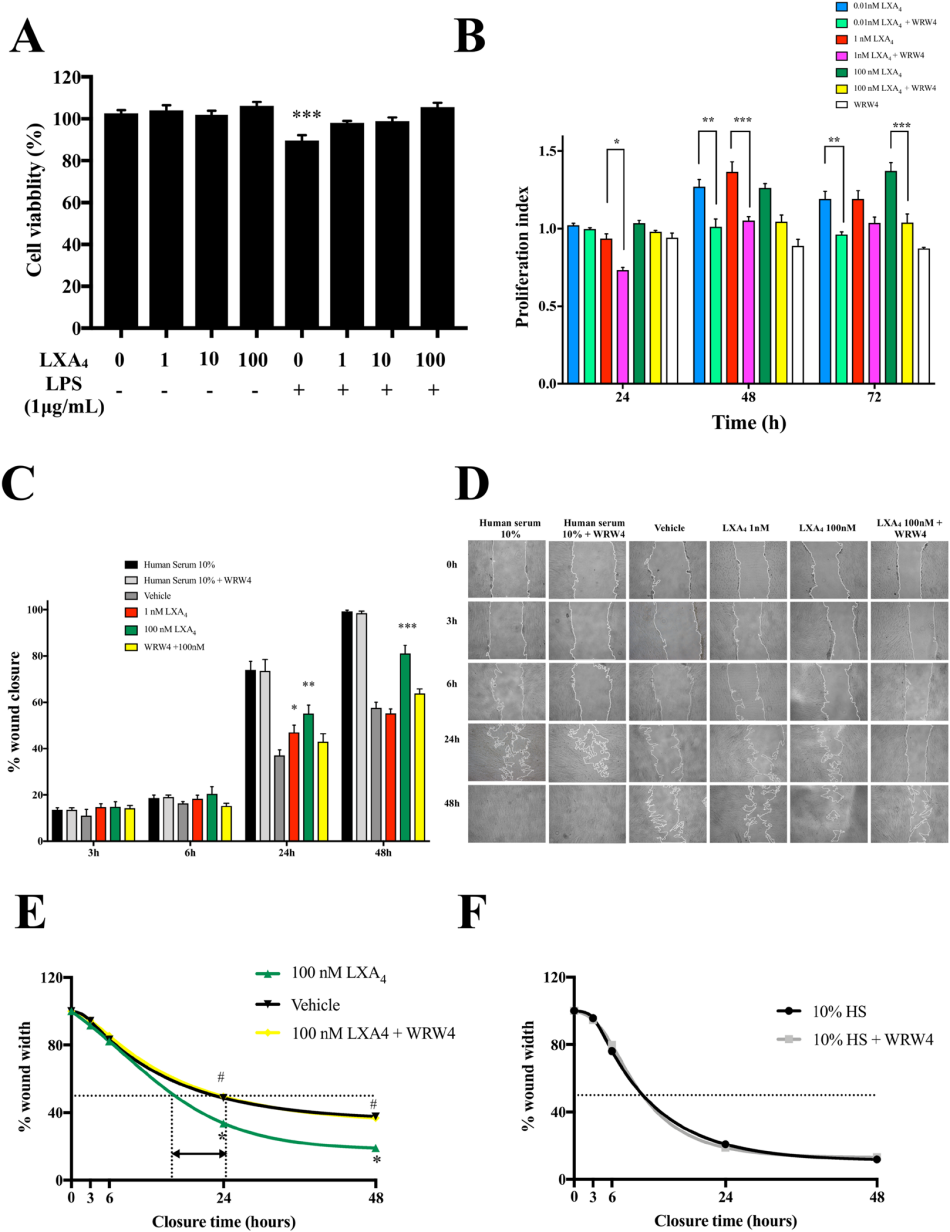
ALX/FPR2 dependent stimulation of SCAP cell viability, proliferation, migration by lipoxin A4 (LXA_4_). (**A**) MTT method was used to analyze the cell viability. Cell viability was reduced by 11% after cells were incubated in LPS (1 μg/mL) for 24 h, and with no effect on the pretreatment of LXA_4_ (1, 10 and 100 nM). ***p < 0.001 versus control. (**B**) Proliferation was evaluated by MTT proliferation test. SCAP exposed to increasing concentrations of LXA_4_ (0.01–100 nM) for 24, 48, and 72 hours displayed a concentration and time-dependent increment in proliferation, with a maximum at 48 and 72 hours.This effect was suppressed by ALX/FPR2 selective antagonist peptide WRW4 (Trp-Arg-Trp-Trp-Trp-Trp-CONH2). Data are expressed as proliferation index (cell number with LXA_4_ per cell number with vehicle). *p < 0.05 WRW4 + 0.01 nM LXA_4_versus 0.01 nM LXA_4_; **p < 0.01 WRW4 + 1 nM LXA_4_ versus 1nM LXA_4_; ***p < 0.001 WRW4 + 100 nM LXA_4_ versus 100 nM LXA_4_. (**C,D**) Analysis of SCAP migration after exposure to LXA_4_ (1 nM and 100 nM) or vehicle. Human Serum 10%, and human serum 10% +WRW4 were uses as positive controls. Migration was evaluated by a wound scratch healing test. Wound closure was quantified at 3, 6, 24, and 48 hours post-wounding, using ImageJ software. *p < 0.05 **p < 0.01 ***p < 0.001 versus vehicle. (D) Representative images of wound closure at 3, 6, 24, and 48 hours (magnification: x4). (**E**) Kinetic to reduce wound closure of SCAP treated with LXA_4_ (100 nM) in comparison with vehicle and with SCAP treated with WRW4 before LXA_4_ (100 nM). *p < 0.05 LXA_4_ vs. vehicle; #p < 0.001 LXA_4_ + WRW4 vs. LXA_4_. (**F**) Effects of WRW4 treatment on Human serum (HS)-induced migration.

Potential cytotoxic effects of LXA_4_ in SCAP were evaluated in the absence or presence of LPS using 3-(4,5-Dimethyl-2-thiazolyl)-2,5-diphenyl-2H-tetrazolium bromide (MTT) assays. LXA_4_ (1, 10 and 100 nM) and a vehicle did not affect cell viability. A viability decrease was observed after application of 1 μg/mL of LPS only (Fig. 3A). Cells pretreated with LXA_4_ for 30 min showed no significant increase in viability compared with cells that were treated with LPS only (Fig. 3A). Because SCAP may play a role in resolution of inflammation, their self-renewal and migration capabilities are fundamental to achieve a pathophysiological impact. Therefore, we determined whether these functions could be modulated by LXA_4_. Proliferation was evaluated by cell count and trypan blue exclusion. At 48 h and 72 h, LXA_4_ in different doses increased proliferation, and this effect was suppressed by the formyl peptide receptor antagonist, WRW4 (Trp-Arg-Trp-Trp-Trp-Trp-CONH2) (Fig. 3B). Then, in an *in vitro* wound healing assay to assess migration of SCAP, we found that 1 nM and 100 nM LXA_4_ significantly augmented migration capacities, accelerating wound closure of SCAP at 24 h and 48 h (Fig. 3C,D). W50 (defined as the time to reduce wound width by 50%) was shortened from 24 to 18 hours when 100 nM of LXA_4_ was used (Fig. 3E). This effect was abrogated by WRW4 (Fig. 3C–E), which, by itself, did not affect W50 of cells exposed to human serum (Fig. 3F).

### LXA_4_ inhibits LPS-induced production of inflammatory cytokines, chemokines and vascular endothelial growth factor (VEGF) by SCAP

**Figure 4:**
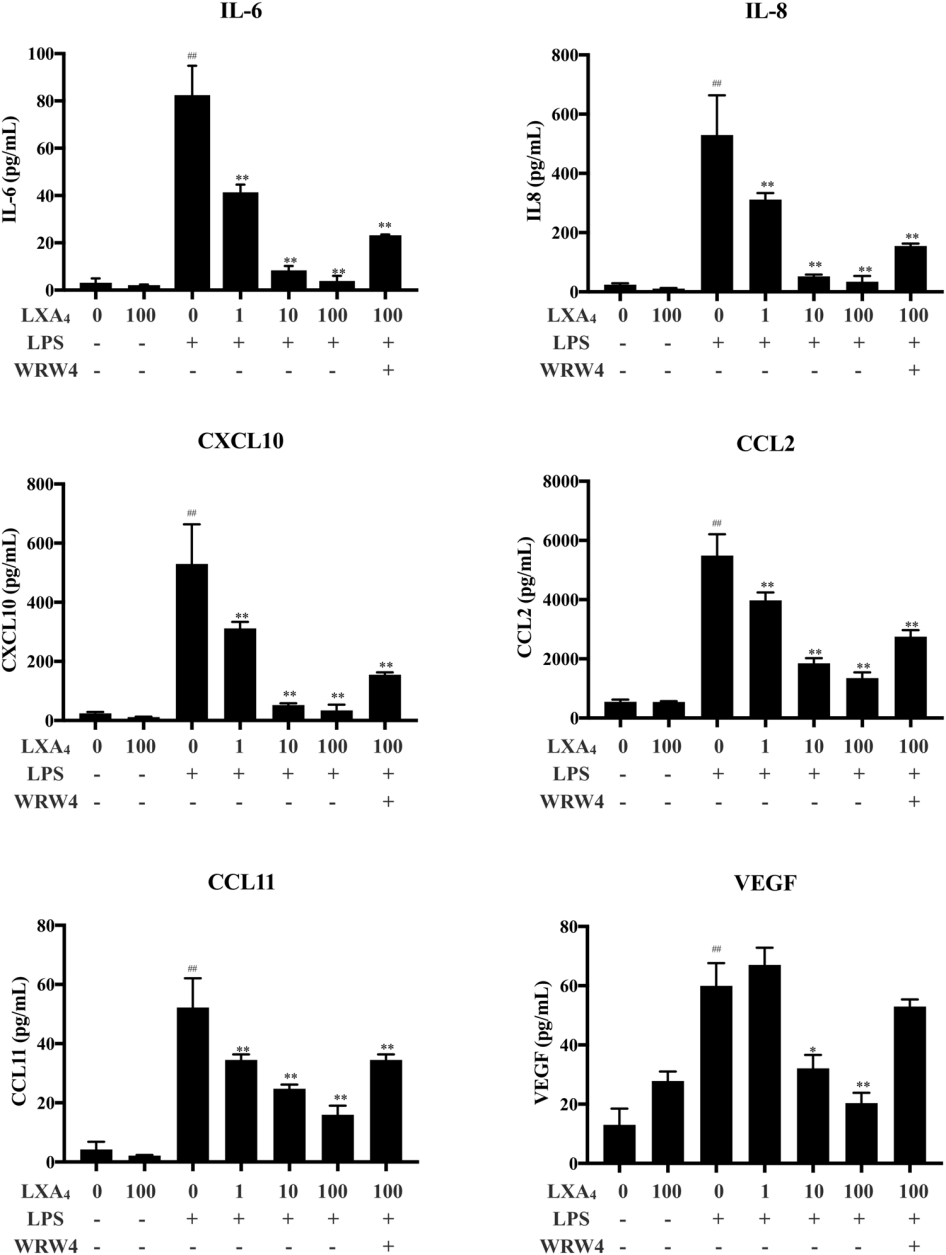
Result of multiplex bead-based assay showing inhibition with dose effect of LXA_4_ on IL-6, IL-8, CXCL10, CCL2, CCL11 and VEGF secretion by SCAP. SCAP were pretreated with vehicle (0.035% ethanol) or various concentrations of LXA_4_ (1, 10 and 100 nM) for 30 min in the absence or presence of WRW4 (30 min before LXA_4_ treatment) followed by stimulation with LPS (1 μg/ml) for 24 h. The concentration IL-6, IL-8, CXCL10, CCL2, CCL11 and VEGF in culture media was measured using a multiplex bead-based assay. **p < 0.01 compared with LPS in the absence of LXA_4_; ##p < 0.01 compared with control cells (vehicle).

To investigate whether the production of inflammatory cytokine IL-6, chemokines (IL-8, CCL2, CXCL10, CCL11) and growth factor VEGF was inhibited by LXA_4_ in SCAPs, multiplex analyses of cytokines and chemokines were performed. Here, SCAP were pretreated with 1 μg/mL LPS for 24 hours and compared with a control group (vehicle only). Stimulation of SCAP with LPS significantly upregulated the secretion of inflammatory cytokine IL-6, chemokines (IL-8, CCL2, CXCL10, CCL11) and of growth factor VEGF in comparison with control conditions. LXA_4_ significantly inhibited the LPS-induced increase in a concentration-dependent manner. To evaluate the role of the ALX/FPR2 in the anti-inflammatory effects of LXA_4_, SCAPs were treated with WRW4 (10 μM, 30 min) prior to treatment with LXA_4_ (100 nM). Pretreatment with WRW4 tended to inhibit these effects in response to LXA_4_, however the difference was not statically significant except for VEGF (Fig. 4).

### LXA_4_ enhances the immunomodulatory potential of SCAPs

**Figure 5:**
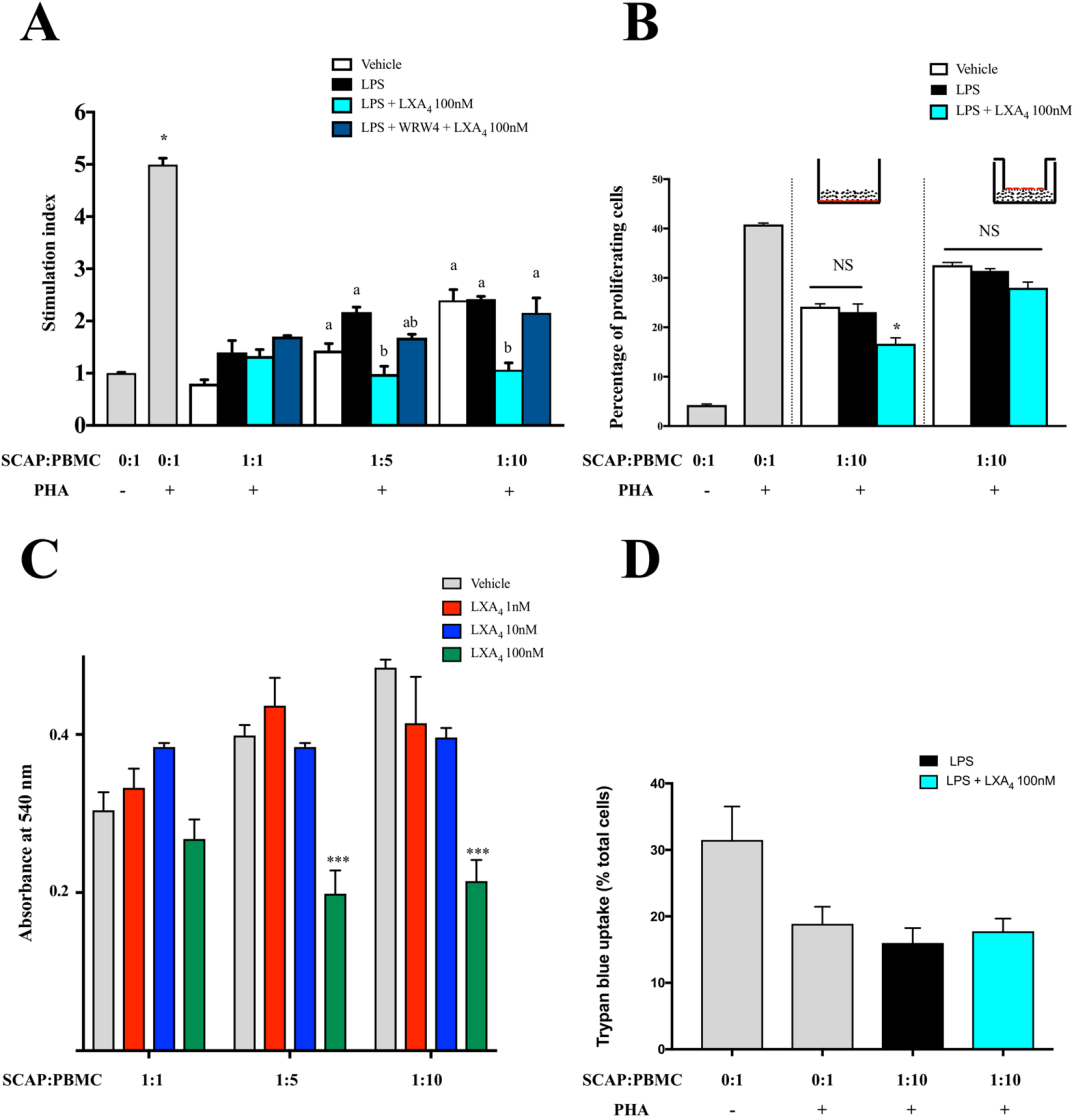
LXA_4_ enhanced immunomodulatory properties of SCAP on PBMC stimulation and proliferation. (**A**) SCAP were first stimulated with LXA_4_ 100 nM or LXA_4_ 100 nM + LPS (1 μg/ml) or WRW4 + LXA_4_ 100 nM + LPS (1 μg/ml) during 24 h. PBMC were stimulated with PHA and co-cultured with SCAP at different ratio (1:1, 1:5, 1:10). After 5 days, Stimulation index was calculated with MTT assay. *p < 0.05. Columns containing the same letter or letters are not significantly different (p> 0.05). (**B**) SCAP (red dots) were first stimulated with LXA_4_ only or LXA_4_ + LPS (1 μg/ml) for 24 h. CFSE-labeled PBMC (solid round cells) were stimulated with PHA in the lower chamber of a Transwell. SCAP were co-cultured with the stimulated PBMC (ratio SCAP/PBMC 1:10) either in direct contact or separated by a Transwell membrane. After 5 days of co-culture, proliferating PBMC were assessed by CFSE staining and flow cytometric analysis. *p < 0.05. (**C**) SCAP were first stimulated with LXA_4_ (0, 1, 10 or 100 nM) during 24 h and co-cultured with (PHA)-stimulated PBMC at different ratio (1:1, 1:5, 1:10). After 5 days, the effect of the different dose of LXA_4_ on PBMC proliferation was assessed by MTT. ***p < 0.001 compared with control cells (vehicle). (**D**) SCAP were first stimulated with LXA_4_ 100 nM or LXA_4_ 100 nM + LPS (1 μg/ml) during 24 h. PBMC were stimulated with PHA and co-cultured with SCAP at different ratio (1:1, 1:5, 1:10). After 5 days, dead cells assessment was calculated by trypan blue uptake. PHA phytohaemagglutinin A, CFSE carboxyfluorescein succinimidyl ester.

We sought to investigate the effect of LXA_4_ on immunomodulatory potential of LPS-stimulated SCAP. SCAP possess low immunogenicity and can also suppress the one-way mixed lymphocyte reaction (MLR) in a dose-dependent manner^13^. First, we sought to investigate the effect of different concentrations of LXA_4_ (0, 1, 10, and 100 nM) on SCAP in MLR. SCAP were co-cultured with phytohemagglutinin (PHA)-stimulated PBMC in different ratios. Pretreatment of SCAP with 100 nM LXA_4_ significantly amplified the suppressive effect on PBMC when 1:5 and 1:10 dilution ratios were used (Fig. 5A).

According to the pro-inflammatory effect of LPS on SCAP population (Fig. 4), LPS may significantly influence the immunomodulatory properties of SCAP on the proliferation of PBMC. Therefore, we then examined the effect of LXA_4_ on SCAP in a mixed leukocyte reaction where SCAP were pre-stimulated with LPS. Proliferation of PHA-stimulated PBMC served as a positive control. The proliferation of PBMC was analyzed using the MTT method. SCAP suppressed proliferation of PHA-stimulated PBMC in a dose dependent manner (Fig. 5B). Pretreatment with LPS (1 μg/mL) did not inhibit the suppressive effect of SCAP on PBMC proliferation. However, pretreatment of SCAP with 100 nM of LXA_4_ significantly increased the suppressive effect on PBMC when 1:5 and 1:10 dilution ratios were used (Fig. 5B). This effect was abrogated by WRW4 (Fig. 5B).

To elucidate whether the immunosuppressive action of LXA_4_ on SCAPs relies on secretion of soluble factors or requires cell-to cell contact, we performed other MLR using transwell inserts with a pore diameter of 0.4 μm. Percentages of proliferating cells were calculated by the carboxyfluorescein diacetate succinimidyl ester dilution method using flow cytometry. We confirmed results shown in Fig. 5B with PBMC diluted in 1:10 ratio. SCAP suppressed PHA-induced PBMC proliferation. LPS (1 μg/mL) did not inhibit the suppressive effect of SCAP on PBMC proliferation. However, we demonstrated that the suppressive action of 100 nM LXA_4_ was efficient only when cells were in direct contact (Fig. 5C).

Finally, to measure viability of PBMC after PHA stimulation, the trypan blue uptake method was used. There was no difference in viability of PBMC (trypan blue uptake) between groups with LPS and LPS + LXA_4_ (Fig. 5D).

## Discussion

The apical papilla of developing teeth represents an enriched source of stem cells. These so-called Stem Cells of the Apical Papilla (SCAP) are considered as a potential source for dental pulp tissue regeneration and seem to be involved in the interplay between the processes of inflammation and regeneration^14^.

In this study, we identified for the first time that SCAP express the lipoxin receptor ALX/FPR2. The expression of ALX/FPR2 is upregulated by a variety of inflammatory stimulants such as TNF-α, interferon-γ as well as ligands for TLR-2, 3, 4, 7 and 9 in different cell types^15,16^. Although the effect was apparently weaker in comparison with microglial cells or microvascular endothelial cells, we demonstrated that important inflammatory mediators of pulpal and periapical diseases such as TNF-α and LPS upregulated ALX/FPR2 expression in SCAP. Further, we demonstrated that the LXA_4_-ALX/FPR2 axis increased proliferation and migration of SCAP. Pretreatment with LXA_4_ abrogated the paracrine activity of SCAP stimulated with LPS. Interestingly, we also showed that LXA_4_ enhanced immunomodulatory properties of SCAP.

Mesenchymal Stem Cells (MSC), SCAP and fibroblasts share common features (for instance their set of cell surface markers is negative for CD45 and positive for CD90, CD105) and they can also be induced to differentiate into adipocytes, chondrocytes and osteoblasts. Moreover, SCAP have immunomodulatory properties similar to MSCs^17^. SCAP were first characterized based on the expression of the surface marker STRO-1^18^. In other experiments STRO-1 sorted SCAP were also compared with CD73, CD90 and CD105-sorted SCAP, with non-purified SCAP and with human gingival fibroblasts for phenotyping and *in vitro* osteogenic differentiation (Supplementary File S11A,C). However, STRO-1 + SCAP represent a small subset of SCAP population and are not representative of SCAP population present in the apical papilla^19^. Therefore we used a classical outgrowth technique for isolation of SCAP^8,20^. As expected, the effect of SCAP passages did not alter *in vitro* differentiation and cell surface markers of the cells (Supplementary File S2), demonstrating the stemness of the unsorted SCAP population.

It has been shown that not all cells from apical papilla are stem cells, *e*.*g*., fibroblasts have been noted^18^. However, in our study, we directly utilized the whole heterogeneous cell population, since there has been no compelling evidence that a purified stem cell subpopulation would be more homogeneous than original population^21^.

LXA_4_ was the first identified endogenous ligand for ALX/FPR2^22^. This receptor has been shown in several cell types, including leukocytes, microglia, endothelial and epithelial cells^23^. Of note, other pro-resolving mediators (resolvins) are also ALX/FPR2 ligands, and both, lipoxins and resolvins, have been used with promising results in periodontal disease and pulpitis models^24,25^.

Only recently, the expression of ALX/FPR2 was described in human Mesenchymal Stem Cells and periodontal ligament cells. In acute lung injury, human MSC promoted the resolution of inflammation and prolonged survival of mice in part through exogenous LXA_4_^26^. We found that SCAP expressed a relatively low level of ALX/FPR2 in comparison with PBMC, however, this expression was upregulated by pro-inflammatory factors (TNF-α and LPS). These results are in line with other studies using microglial or endothelial cells^16,27^. Although no mechanistic studies have been performed at this point, we may hypothesize that ALX/FPR2 is upregulated through c-Jun N-Terminal protein kinase and transcription factor NF-κB signaling pathways as discussed in other studies^16^.

Cell proliferation and migration are critical processes in regeneration of connective tissue and, in particular, of dental pulp and dentin. In our study, we demonstrated that the LXA_4_-ALX/FPR2 axis was involved in SCAP proliferation and migration. This result is consistent with earlier studies showing the role of the formyl peptide receptor in cell migration and proliferation of stem cells^28,29^. The effect of chemotactic receptors/ligands to facilitate SCAP recruitment has already been explored. For instance, SCAP can be chemo-attracted by the Stromal Cell-derived Factor-1α/ chemokine receptor 4 axis^30^. We may speculate that the recruitment and expansion of SCAP could be further modulated by LXA_4._

To date, there is only limited data regarding the behavior of SCAP in an inflammatory context. Because of the presence of Gram-negative bacteria in the pulp space of infected teeth, LPS from Gram-negative bacteria has been used to stimulate toll-like receptor 4^31^. Interestingly, in dental follicle cells, only LPS from *Escherichia coli*, but not from *Porphyromonas gingivalis*, induced expression of cytokines and chemokines^32^. In our study, we used LPS from *E*. *coli*. LPS markedly increased production of inflammatory cytokines, chemokines and vascular endothelial growth factor (VEGF) by SCAP. In another study, LPS from *E*. *coli* induced production of pro-inflammatory cytokines and chemokines including IL-6, IL-8, and TNF-α in SCAP^33^. We have shown here that LXA_4_ reduced LPS-induced production of cytokines, chemokines and VEGF in a dose dependent manner with the maximum effect of 100 nM LXA_4_. In microglial cells or corneal fibroblasts, comparable concentrations of LXA_4_ were used and inhibited pro-inflammatory cytokine such as IL-1β, ΤΝF-α as well as nitric oxide production in a concentration-dependent manner^27^. This inhibitory effect of LXA_4_ on LPS-induced inflammatory mediators was ALX/FPR2 mediated, since it was attenuated when SCAP were pretreated with the formyl peptide receptor antagonist WRW4 (Trp-Arg-Trp-Trp-Trp-Trp-CONH2). These results are in agreement with data from ALX/FPR2^−/−^ mice, where LPS treatment showed an increase in pro-inflammatory cytokines^28^.

However, WRW4 did not completely block the action of LXA_4,_ in particular in response to IL-6 and IL-8. WRW4’s action is believed to be through inhibition of agonist binding to ALX/FPR2, and inhibition of intracellular calcium release^34^. Moreover, WRW4 can block the activation of ERK1/2 and p38 MAPK signaling^35^. Although lipoxins and epi-lipoxins exert their anti-inflammatory effects through signals generated by binding to ALX/FPR2, lipoxins have also been found to interact with other receptors like G protein-coupled receptor 32 (GPR 32)^7^, aryl hydrocarbon receptor^36^, and high affinity cysteinyl leukotriene receptor^37^. Interestingly, human mesenchymal stem cells express both the cysteinyl leukotriene type 1 receptor and the aryl hydrocarbon receptor. These receptors, upon positive activation, may potentially regulate the MSC-associated immunomodulatory function in particular secretion of cytokines such as IL-6 and IL-8^38^.

Only limited data exists regarding the effect of lipoxins on adaptive immune cells; one study described that LXA_4_ decreased IgM and IgG production by activated human B cells through ALX/FPR2-dependent signaling^6^. Recent reports suggested that dental mesenchymal stem cells, in particular SCAP, could suppress mitogen- or allogenic-stimulated proliferation of PBMC or T-cells *in vitro* at a ratio SCAP/PBMC higher than 1/10^39–41^. A lower mesenchymal stem cell number in culture was non-suppressive^42,43^. Therefore, aiming to see the immunomodulatory effect of SCAP *in vitro* with conditions that could mimic *in vivo* situation, we decided to study the following SCAP:PBMC ratio 1:10; 1:5 and 1:1. In our study, SCAP suppressed phytohemagglutinin-stimulated PBMC proliferation in a dose-dependent manner. Moreover, this inhibitory effect was increased by LXA_4_ pretreatment and was also observed when SCAP and PBMC were in direct contact. Such a modulation of the pulpal inflammatory response has been suggested as a way for optimizing endodontic therapeutics^44^.

Taken together, our data demonstrate that LXA_4,_ a potent endogenous signal involved in the resolution phase of inflammation, can modulate the behavior of stem cells in inflammatory conditions *in vitro*. Our findings may help to elucidate mechanisms, by which stem cells can influence the outcome of inflammatory processes and disorders. We demonstrated that ALX/FPR2 receptor is essential for recruitment, proliferation and immunomodulatory functions of SCAP. Thus, ALX/FPR2 receptor may represent a novel molecular target for development of new drugs for therapies that require enhancement of stem cell-mediated pro-resolving functions.

## Materials and Methods

### Isolation, Culture, and Characterization of SCAP and PDLC

Apical papilla tissue was collected from third molars extracted from medically healthy patients (16 to 25 years old); informed consent had been obtained in accordance with a protocol approved by the Institutional Review Board at the Arthur A. Dugoni School of Dentistry at the University of the Pacific (IRB protocol #16-128). SCAP culture was established as previously described^45^. Similarly, periodontal ligament cells (PDLC) were obtained from scraped PDL tissue. Cells were cultured in alpha minimum essential medium (α-MEM) supplemented with 1% L-glutamine, 1% penicillin/streptomycin/amphotericin B (all ThermoFischer Scientific, Pittsburgh, PA, USA) and 10% human serum (HS) (Sigma-Aldrich, St Louis, MO, USA). SCAP and PDLC at passage 4 were analyzed for cell surface antigen expression by flow cytometry using Guava easyCyte 8HT flow cytometer (EMD Millipore, Billerica, MA, USA). Fluorochrome-conjugated monoclonal mouse anti- human against CD45-APC/Cy7, CD90-PE-CY7, CD105-APC, CD146-PerCP/Cy5.5 and STRO-1- FITC or isotype controls (all from BioLegend, San Diego, USA) were used. Data were analyzed using InCyte 2.5 software (EMD Millipore). Cells counts were performed at each passage, and the population doubling times (PDTs) were calculated.

### *In vitro* differentiation assays

To show a multipotentiality, osteogenic and chondrogenic pathways of differentiation were evaluated using alizarin red S and alcian blue, respectively (Sigma-Aldrich). Briefly, cells were plated in 12-well plates at 30,000 cells/well and cultured for 21 days with the appropriate media being replaced every 2–3 days.

### LPS, TNF-α application

When the cells reach 80%–90% confluence, they were incubated in starvation medium (1% HS) for 12 hours. Then, cells were treated with various concentrations of *Escherichia coli* O111:B4 Lipopolysaccharide (LPS) (Sigma-Aldrich) and/or various concentrations of Recombinant Human TNF-α (R&D Systems, Minneapolis, USA).

### Analysis of ALX/FPR2 Expression

#### Flow cytometry

Surface and intracellular expression of the lipoxin receptor, ALX/FPR2, was evaluated in PDLC and SCAP from different donors using a monoclonal anti-ALX/FPR2 primary antibody (Thermo Fischer Scientific) and incubated with an anti-mouse PE-conjugated secondary antibody (BioLegend). For intracellular staining, cells were permeabilized with Tween-20 (Thermo Fischer Scientific). Secondary antibody-matched controls were used to assess unspecific fluorescence.

#### Immunofluorescence and Confocal Microscopy

SCAP and PDLC grown on glass coverslips were fixed with 4% paraformaldehyde, permeabilized with 0.2% Tween-20 for 20 minutes, and neutralized with 1% bovine serum albumin (BSA) (Affymetrix, Santa Clara, CA, USA) for 30 minutes. Incubation with primary mouse anti-ALX/FPR2 antibody (1:500) was followed by exposure to secondary FITC conjugated goat anti-Mouse (BioLegend) (1:1000). After washing in PBS, samples were stained with ProLong® Gold Antifade Mountant with DAPI (Thermo Fischer Scientific). Samples were examined with a Leica TCS-SPE II confocal laser-scanning microscope (Leica, Mannheim, Germany) at 10X and 43X original magnification. Images were acquired with LAS AF V.3 software (Leica).

#### Cells-to-C_T_ 1-Step quantitative RT-PCR

SCAP stimulated with LPS (1 μg/mL) for 24 h were lysed using the Cells-to CT™ 1-Step TaqMan® Kit (Thermo Fischer Scientific). The resulting lysate was then used for one-step real-time RT-PCR with a TaqMan® gene expression assay for ALX/FPR2 (assay Hs00265954_m1), and with glyceraldehyde 3-phosphate dehydrogenase as an endogenous reference (assay Hs02786624_g1), using a StepOnePlus™ apparatus (Applied Biosystems). The relative ALX/FPR2 gene expression was determined using a comparative delta-delta cycle threshold method (DDCt) with a control group as a calibrator^46^.

### MTT Analysis for Cell Viability

SCAPs were seeded in 96-well plates at a density of 7 × 10³ cells, pretreated with different concentrations of LXA_4_ (Cayman Chemical, Ann Arbor, MI, USA) for 30 min, and incubated with or without LPS (1 μg/mL) for 24 h in the continued presence of LXA_4_. Wells containing only culture medium only served as controls for non-specific dye reduction. Then, the medium was removed and cells were incubated with 0.5 mg/mL of 3-(4,5-Dimethyl-2-thiazolyl)-2,5-diphenyl-2H-tetrazolium bromide (MTT) (ThermoFischer Scientific) in full medium at 37 °C, 5% CO2. After 4 hours of incubation at 37 °C, the medium was removed and the formazan crystals dissolved in DMSO (Sigma-Aldrich). Absorbance was measured at 540 nm using a VersaMax™ Microplate Reader. The results were expressed as the percentage of surviving cells compared to control cells.

### Proliferation Assay

SCAP (2 × 10^3^ cells per well) were seeded in 96-well microplates with complete medium overnight. The following day, cells were exposed to LXA_4_ (0.01 to 100 nM), the formyl peptide receptor antagonist WRW4 (Trp-Arg-Trp-Trp-Trp-Trp-CONH2) (R&D Systems) (10 µM, for 30 minutes) before LXA_4_ or vehicle (0.035% ethanol) diluted with 1% HS α-MEM. Vehicle controls were defined as 1 × PBS with 0.035% ethanol by volume, equivalent to the highest concentration of lipoxins used in the experiment (100 nM) LXA_4._ Cells were enumerated after 24, 48, and 72 hours using trypan blue exclusion test. Proliferation was calculated as ratio between LXA_4_-and vehicle-treated SCAPs. LXA_4_-affected cellular proliferation index was calculated as ratio between LXA_4_ and WRW4 + LXA_4_-treated cells.

### *In Vitro* Wound Healing Assay

SCAP (5 × 10^4^ cells per well) were grown in 24-well plates in complete medium until 80% to 90% confluency was reached. A disposable plastic pipette tip (200 μl) was used to make a scratch across the monolayer of cells. Cell debris was rinsed away by medium and increasing concentrations of LXA_4_, with or without WRW4 were added to each well. SCAPs maintained in complete medium served as a control of the maximal healing capacity. Cell wounds were monitored at 0, 3, 6, 24, and 48 hours using a phase contrast microscope equipped with a digital camera. Each well was photographed at four locations along the original wound boundary and wound width was quantified using ImageJ software.

### Multiplex analysis of cytokines and chemokines

SCAPs were seeded in 24-well plates at a density of 5 × 10^4^ cells, pretreated with different concentrations of LXA_4_ and/or WRW4 for 30 min, and incubated with or without LPS (1 μg/mL) for 24 h, then, culture media were harvested. Cytokines and chemokines were analyzed with the HCYTMAG-60K-PX41 Milliplex kit (EMD Millipore). The procedure described in the manufacturer’s manual was followed. Plates were analyzed using a Magpix platform (Luminex) equipped with the xPONENT 3.1 software and the concentration of each cytokine was calculated from raw data. Analyte concentrations were determined by a five-parameter logistic curve.

### Mixed leukocyte reactions (MLR)

SCAP 5 × 10^4^ were seeded in wells of a 24-well plate or into a transwell inserts for 24-well plates (0.4 μm) (Corning, NY, USA) in RPMI medium +10% HS (ThermoFischer Scientific). SCAP were stimulated with LPS (1 μg/mL) for 24 h, before application of LXA_4_ (1 to 100 nM), and/or WRW4 (10 μM), while control SCAPs were left untreated. SCAP were washed twice in PBS with 10% Human Serum, treated with mitomycin C (Sigma-Aldrich) at the final concentration of 25 μg/mL for 30 min, and then washed 4 times with PBS + 10% Human Serum. Human peripheral blood mononuclear cells (PBMC) (AllCells, Alameda, CA, USA) in dilution ratios 1:1, 1:5 and 1:10 were stimulated with 5 μg/mL phytohemagglutinin (PHA) (Sigma-Aldrich) and immediately added to the wells containing SCAP. Negative controls (PBMC only) and positive controls (PBMC + PHA) were used. Results of MLR were evaluated after five days of cultivation.

### MTT assay for stimulation index of PBMC

The proliferation of PBMCs was analyzed using the MTT method. Stimulation index (SI) values were calculated by the following formula: SI = proliferation of stimulated PBMC with or without SCAP/proliferation of unstimulated PBMC alone^47^.

### CFSE staining to estimate percentage of proliferating PBMC

PBMCs were labeled with 2 µM carboxyfluorescein diacetate succinimidyl ester (CFSE) (ThermoFischer Scientific) before being added to the mixed cell culture. Percentage of proliferating cells was calculated by the CFSE dilution method using flow cytometry.

### Trypan blue uptake for measurement of PBMC viability

After 5 days of MLR, PBMCs were harvested and suspended in 0.4% trypan blue. Dead cells (colored by trypan blue), were counted and the percentage of positive cells to total cells was calculated.

### Statistical Analysis

All results were expressed as the means ± standard error of the mean (SEM) of triplicate measurements; all experiments were repeated at least 3 times, except for multiplex analysis of cytokines which were done in duplicates. Data were analyzed and visualized using GraphPad Prism 7.0. Statistical analyses were performed using Student’s t tests or 1-way analysis of variance (ANOVA) followed by Dunnett post-tests. *P* < 0.05 was regarded as statistically significant.

## Electronic supplementary material

Supplemental Material
