# Supplementary material for: Lipoxin A4 Attenuates the Inflammatory Response in Stem Cells of the Apical Papilla via ALX/FPR2
Source: Sci Rep. 2018 Jun 11;8:8921. doi: 10.1038/s41598-018-27194-7 (PMC5995968; doi:10.1038/s41598-018-27194-7)
Supplement: Supplementary file 1 — Supplemental Material [file 41598_2018_27194_MOESM1_ESM.pdf]

Supplemental Material

Lipoxin A_4 Attenuates the Inflammatory Response in Stem Cells of the Apical Papilla via ALX/FPR2

Gaudin A*[1,2], Tolar M[3], Peters OA[4,5]

[1]Department of Endodontics, University of Nantes, Nantes, France

[2]Centre de Recherche en Transplantation et Immunologie UMR1064, INSERM, Université de Nantes, France

[3]Department of Orthodontics, University of the Pacific School of Dentistry, CA, San Francisco, USA

[4]Department of Endodontics, University of the Pacific School of Dentistry, CA, San Francisco, USA

[5]University of Queensland, School of Dentistry, Oral Health Centre, Herston, QLD, Australia

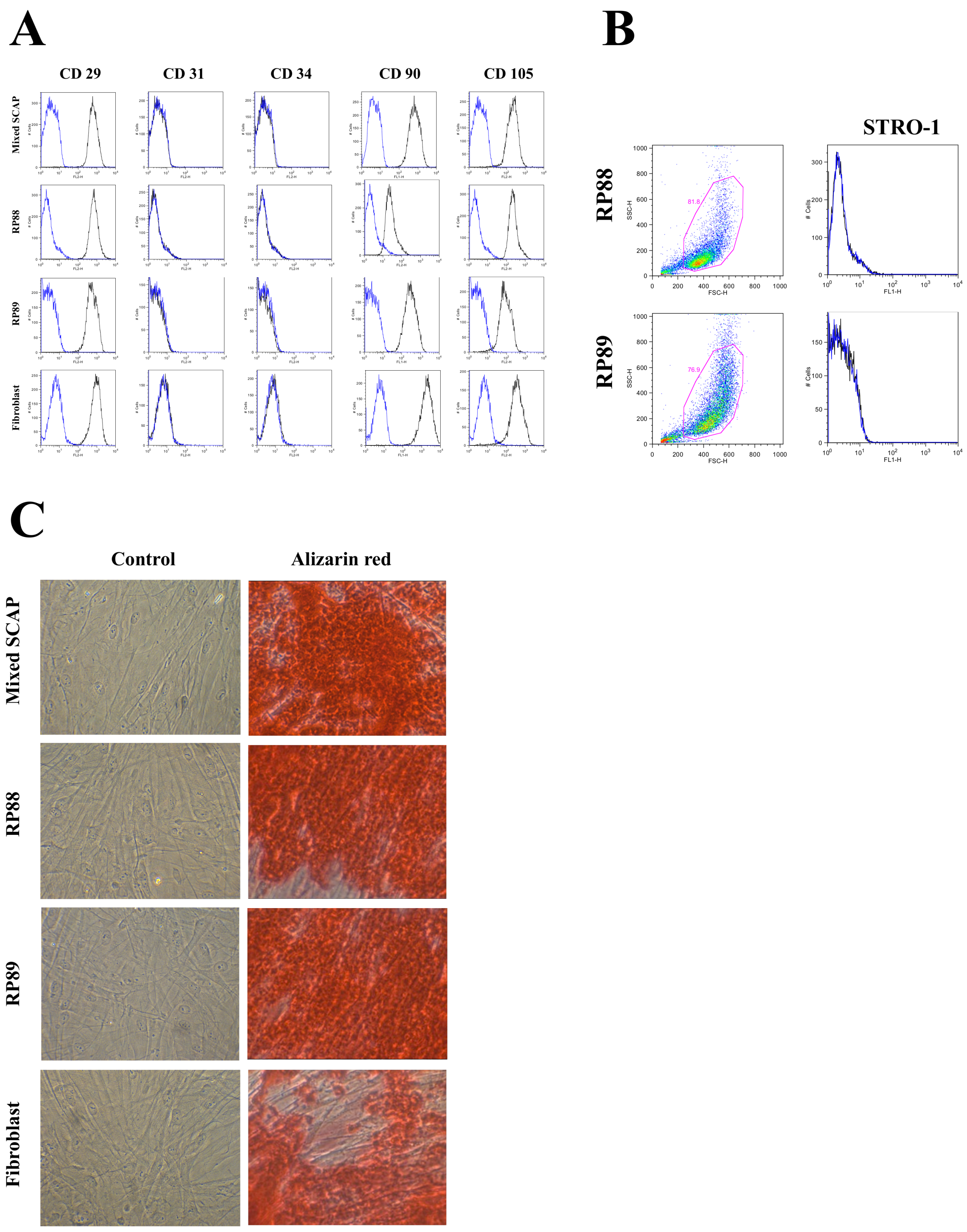

Supplemental Figure 1. Characterization of mixed and purified stem cells from the apical papilla in comparison with gingival fibroblast. **(A)** Flow cytometry analysis of representative histograms showed that mixed SCAP, purified SCAP RP88 and RP 89, and human gingival fibroblast expressed cell surface human mesenchymal stem markers (CD90, CD105, CD29) and lacked the expression for (CD31 and CD34) (in black) compared with their appropriate isotype controls (blue line). Mixed SCAP (isolation method was outgrowth technique without cell purification), RP88 (isolation method was enzymatic digestion, and SCAP were purified with immunomagnetic separation using STRO-1 as cell purification marker, gift by Dr. Anibal Diogenes, University of Texas (San Antonio, TX, USA))**,** RP89 (isolation method was enzymatic digestion, and SCAP were purified with immunomagnetic separation using CD73, CD90 and CD105 as cell purification markers, from University of Texas (San Antonio, TX, USA))**,** human gingival fibroblast (isolation method was enzymatic digestion, without cell purification, from ATCC (Manassas, MA, USA)). **(B)** Flow cytometry analysis of representative histograms showed that purified SCAP RP88 and purified SCAP RP 89 lost Stro-1 expression. A representative example of the gating strategy on total cells (panel on the left) and Stro-1 (black line) compared with its appropriate isotype controls (blue line). **(C)** Differentiation of mixed SCAP, purified SCAP RP88 and RP 89, and human gingival fibroblast to odontoblast-like. Cells were subjected to differentiation media for 2 weeks, which resulted in deposits positive for alizarin red.

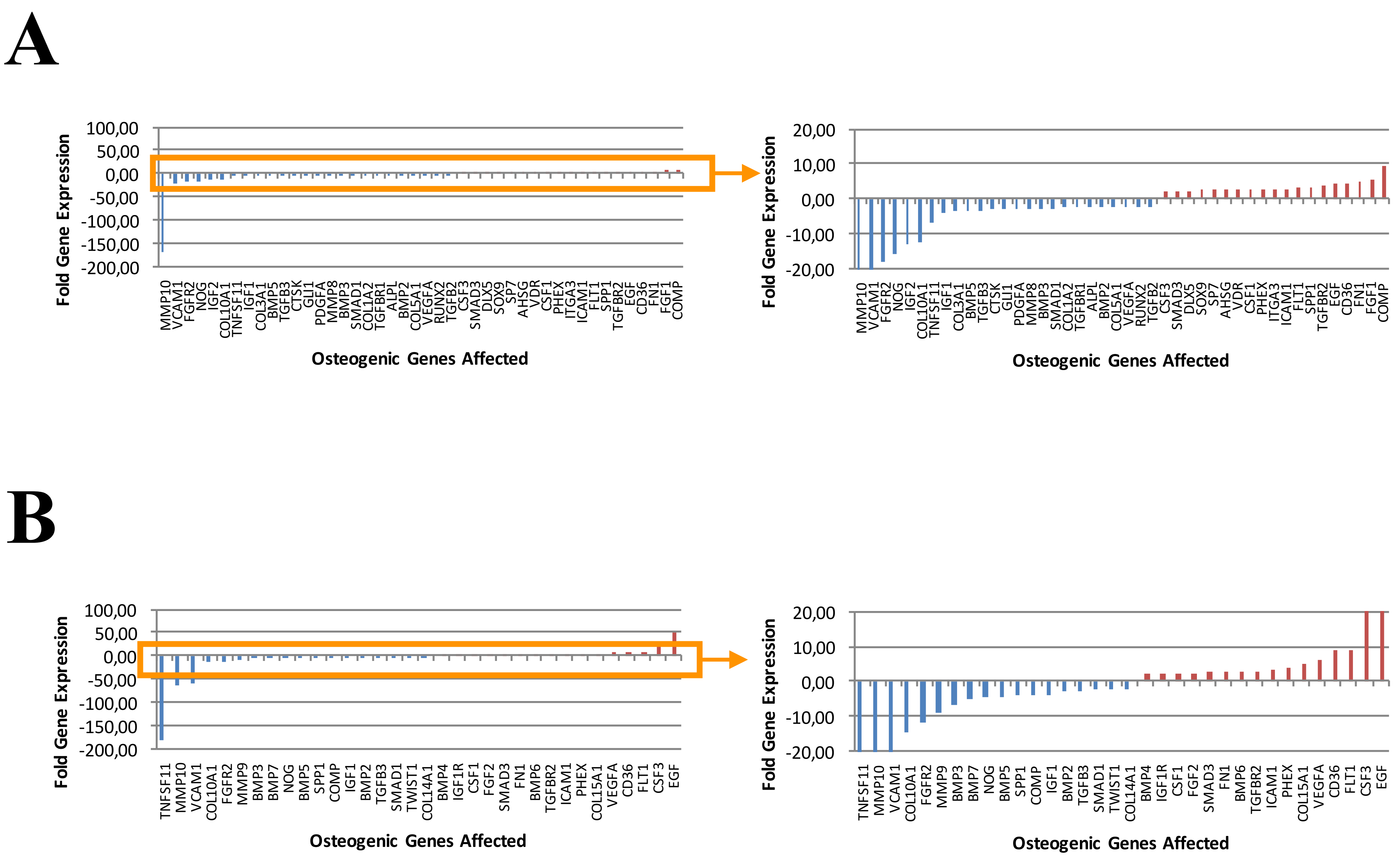

Supplemental Figure 2. Osteogenic gene expression of late relative to early passage of SCAP**.** Real-Time PCR analysis of expression of 84 osteogenic genes (some involved also in chondrogenesis) was performed on osteogenically induced cells and undifferentiated cells, for comparison. **(A)** Undifferentiated cells (SCAP). **(B)** Conventional osteogenic differentiated SCAP. Blue bars indicate increased expression of early relative to late passage cells. Red bars indicate increased expression of late relative to early passage cells.
